# Paying attention to orthography: a visual evoked potential study

**DOI:** 10.3389/fnhum.2013.00199

**Published:** 2013-05-21

**Authors:** Anthony T. Herdman, Osamu Takai

**Affiliations:** BRANE Lab, School of Audiology and Speech Sciences, University of British ColumbiaVancouver, BC, Canada

**Keywords:** orthography, visual evoked potential (VEP), attention, reading, dipole modeling

## Abstract

In adult readers, letters, and words are rapidly identified within visual networks to allow for efficient reading abilities. Neuroimaging studies of orthography have mostly used words and letter strings that recruit many hierarchical levels in reading. Understanding how single letters are processed could provide further insight into orthographic processing. The present study investigated orthographic processing using single letters and pseudoletters when adults were encouraged to pay attention to or away from orthographic features. We measured evoked potentials (EPs) to single letters and pseudoletters from adults while they performed an orthographic-discrimination task (letters vs. pseudoletters), a color-discrimination task (red vs. blue), and a target-detection task (respond to #1 and #2). Larger and later peaking N1 responses (~170 ms) and larger P2 responses (~250 ms) occurred to pseudoletters as compared to letters. This reflected greater visual processing for pseudoletters. Dipole analyses localized this effect to bilateral fusiform and inferior temporal cortices. Moreover, this letter-pseudoletter difference was not modulated by task and thus indicates that directing attention to or away from orthographic features did not affect early visual processing of single letters or pseudoletters within extrastriate regions. Paying attention to orthography or color as compared to disregarding the stimuli (target-detection task) elicited selection negativities at about 175 ms, which were followed by a classical N2-P3 complex. This indicated that the tasks sufficiently drew participant's attention to and away from the stimuli. Together these findings revealed that visual processing of single letters and pseudoletters, in adults, appeared to be sensory-contingent and independent of paying attention to stimulus features (e.g., orthography or color).

## Introduction

Single-letter perception is a prerequisite to word perception and research is starting to unravel the mystery of how the brain processes such basic building blocks of literacy. Reaction times to letters are faster than to symbols or pseudoletters indicating that somewhere along the visual processing stream familiar letters are processed faster (LaBerge, 1973; Herdman, 2011). This might be caused by increased neural activity to letters or faster responding neural ensembles. Evidence for increased neural activity comes from previous neuroimaging research that showed visual evoked responses between 140–190 ms were larger to letters as compare to symbols or pseudoletters (Miller and Wood, 1995; Eulitz et al., 1996; Tarkiainen et al., 1999; Pernet et al., 2003, 2005; Maurer et al., 2005, 2008; Wong et al., 2005; Appelbaum et al., 2009). A negative response recorded from left inferior temporal cortices, termed the N200, has also been shown to be larger for words than for faces or objects (Nobre et al., 1994). However, later responses between 200 and 400 ms were shown to be greater for pseudoletters than letters (Miller and Wood, 1995; Wong et al., 2005; Herdman, 2011). Such processing advantages for letters have been suggested to be a result of language-dominant networks within the left inferior temporal cortices used for word reading (Miller and Wood, 1995; Eulitz et al., 1996; Tarkiainen et al., 1999; McCandliss et al., 2003; Pernet et al., 2003, 2005; Cohen and Dehaene, 2004; Flowers et al., 2004; James et al., 2005; Maurer et al., 2005, 2008; Wong et al., 2005; Joseph et al., 2006). Conversely, a few other studies showed consistently early visual processing differences between letters and pseudoletters across bilateral visual cortices with a possible right-hemispheric dominance (Appelbaum et al., 2009; Herdman, 2011). This provides evidence that orthographic processing is recruiting more bilateral networks, as has been previously proposed (Tagamets et al., 2000). Correspondingly, an fMRI study contrasting false-font strings with words or word-like characters showed a greater signal change in the left inferior temporal regions to words than false-font strings but conversely greater signal change in the right hemisphere to false-font strings than words (Vinckier et al., 2007). The authors suggested that false-font strings might capture greater attention because they are unfamiliar objects and thus recruit more resources within extrastriate regions. This is in line with our previous proposal that pseudoletters elicit prolonged processing within the right extrastriate regions (Herdman, 2011). Furthermore, modulation of neural activity associated with orthographic processing is consistent with findings from Ruz and Nobre (2008) showing that attention to orthography modulated early N200 to words more so than attention to phonology or semantics. However, the attention-related modulation of ERP differences between words and false-font strings were not reported in that study and thus it is difficult to interpret how attention might modulate processing differences between letters and pseudoletters. The current study addressed this issue by manipulating attention toward or away from orthographic features of single letters and pseudoletters.

As compared to the neuroimaging literature on word processing (for reviews see Price, 2000; McCandliss et al., 2003; Price and Delvin, 2003; Cohen and Dehaene, 2004; Dehaene et al., 2005; Maurer et al., 2005, 2008; Grainger et al., 2008), the literature on single-letter processing is less well-developed (e.g., Miller and Wood, 1995; Tarkiainen et al., 1999; James et al., 2005; Wong et al., 2005; Grainger et al., 2008; Appelbaum et al., 2009; Herdman, 2011). Initial stages of reading acquisition are dependent on single-letter recognition (e.g., grapheme-to-phoneme encoding) and thus it is important to understand how the human brain processes individual letters. Interpretations of low-level orthographic processing have mainly been inferred from studies investigating orthography in tasks involving word and letter-string recognition (Grainger et al., 2008). These tasks likely prime neural networks associated with word recognition, such as the visual word form system that could potentially recruit additional processes beyond low-level orthographic processes. For instance, participants are faster at identifying letters in words than when presented alone, commonly known as the word superiority effect (Reicher, 1969; McClelland and Rabinovitch, 1981). Thus, tasks that compare words to letter strings might be recruiting hierarchical processes beyond that of single-letter processing. Evidence for extra processing can be seen in ERP recordings to words or letter strings as compared to single letters in that character strings elicited broader N1 responses as compared to single characters (Wong et al., 2005). Measuring neural responses to single-letters would provide further information about the underpinnings of low-level orthographic processing.

The inconsistent findings for orthographic-related processing within the literature might be due to differences in attention demands on stimulus features as driven by task set or stimulus familiarity (letters vs. pseudoletters). For instance, target-detection tasks that asked participants only to respond after a target (e.g., Appelbaum et al., 2009) might have minimally activated the networks responsible for orthographic processing as compared to tasks that asked participants to discriminate between letters and pseudoletters on a trial-by-trial basis (e.g., Herdman, 2011). Attention is likely less focused on the orthographic stimuli during target-detection tasks than orthographic-discrimination tasks. Reduced attention to a stimulus feature, such as color, is known to modulate early visual processing as evidenced by an early selection negativity (SN) between 140 to 180 ms when attending to stimulus color (Hillyard and Anllo-Vento, 1998). Whether such attention to stimulus feature modulates early orthographic processing differences needs further research. Thus, we investigated the hypothesis that tasks encouraging participants to directly pay attention to orthographic features would enhance early orthographic processing differences between letters and pseudoletters (Herdman, 2011), as compared to tasks that did not encourage recruitment of orthographic networks, such as a color discrimination task or a non-orthographic target-detection task. Contrarily, letters become highly consolidated and relevant for adults who have gained a large amount of experience with these familiar visual objects. Thus, early orthographic processing within the lower-visual centers might be automatic and not task dependent. If this alternative hypothesis is correct then there will be little, if any, change in the early orthographic processing differences between letters and pseudoletters due to directing attention to or away from orthographic features. We used evidence from visual evoked potentials among three tasks (orthography discrimination, color discrimination, and target detection) to determine whether early visual processing of letters and pseudoletters are modulated by paying attention to orthographic features.

## Materials and methods

### Participants

Fifteen right-handed participants (age 18–28 years; 8 female) volunteered for this study. Participant's handedness was determined by Edinburgh Handedness Inventory (Oldfield, 1971). Due to insufficient ERP trials (<40) after artifact rejection of EEG artifacts, datasets from four participants were excluded from this study. All participants disclosed that they had no known sensory or cognitive impairments. Participants were screened for normal 20–20 visual acuity (with corrected lenses) and for color blindness. Informed consent was signed by all participants. This study was approved by the Research Ethics Board at Simon Fraser University, Canada. The experiment lasted for approximately 50 min, consisting of 15–20 min for electrode set-up and 30 min for ERP recording. Participants received a $10 honorarium.

### Stimuli and task

**Figure 1:**
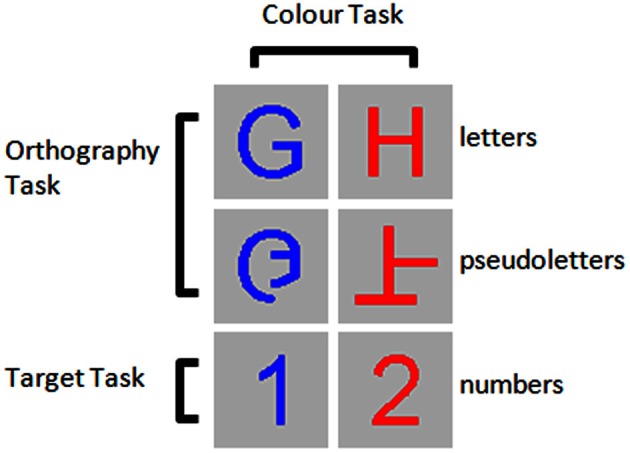
**Example stimuli for the three tasks performed.** Participants were asked to detect numbers 1 and 2 presented among letters and pseudoletters for the Target task, to discriminate between letters and pseudoletters for the Orthography task, to discriminate between red and blue colored stimuli for the Color task.

Visual stimuli were upper-case, roman-alphabetic letters (A, B, D, E, G, H, J, N, P, R, T, U, and Y), pseudoletters (mixed line forms of the letters: A, B, D, E, G, H, J, N, P, R, T, U, and Y), and numbers (1 and 2) presented as red or blue characters on a gray background (Figure 1). Stimuli covered 60 × 60 pixels at the centre of a 19″ VGA monitor with a resolution of 600 × 800 pixels situated approximately 70 cm in front of the participant's eyes. Stimuli were randomly presented for a duration of 500 ms in the central visual field. Stimuli were followed by a black fixation dot on the gray background shown for a random duration between 1500 and 2000 ms. Presentation software (NeuroBehavioral Systems Inc., Albany, CA) was synchronized to the VGA monitor's refresh rate in order to accurately synchronize the stimulus onset with the trigger pulse that was sent to the EEG recording computer.

Participants performed three tasks in separate randomly-assigned blocks. A participant was asked to press one of two buttons with his/her right hand to discriminate between letters and pseudoletters (Orthography Task), to discriminate between red and blue stimuli (Color Task), and to detect target numbers 1 and 2 (Target Task). For the Orthography and Color tasks, 200 letters and 200 pseudoletters were randomly presented across three blocks of 133, 133, and 134 trials with each block lasting about 5 min. Participants were given approximately 30 s of rest between blocks. For the Target task, 200 letters, 200 pseudoletters, and 50 targets (25 number “1” and 25 number “2”) were randomly presented across three blocks of 150 trials with each block lasting about 5 min. Participants were given approximately 30 s of rest between blocks. For the Target task, participants were asked to detect when a number 1 or 2 appeared on the screen by pressing only one button and to ignore the other stimuli (i.e., letters and pseudoletters). Participants were asked to press buttons as accurately and as fast as possible. This allowed us to collect behavioral response accuracy and reaction times to stimuli when button presses were required.

### Data acquisition

EEG was collected using a 136-channel BIOSEMI system (BIOSEMI, www.biosemi.com). Scalp electrodes (128 channels) were situated within a cap in a modified 10–5 configuration with two additional mastoid electrodes (M1 and M2), two inferior occipital electrodes (SI3 and SI4), and four electrooculogram electrodes (SO1, IO1, LO1, and LO2). EEG was amplified and sampled at a rate of 1024 Hz with a band-pass filter of 0.16–256 Hz. For online collection, the 136-electrodes were referenced to a common electrode placed between CPz and CP2. For offline analyses, the 132 scalp-electrodes (excluding electrooculogram channels) were re-referenced to their average reference.

### Data analyses

#### Behavioral

Behavioral accuracy and reaction times were determined from the participants' button presses for each task. Trials with correct button presses within the post-stimulus interval of 100–1500 ms were used to calculate accuracy and reaction times. Correct responses (hits) were correct button presses to corresponding stimulus type (letters and pseudoletters) for the Orthography task, correct button presses to stimulus color (red and blue) for the Color task, and correct button presses to numbers (1 or 2) for the target task. False alarms were considered as incorrect button responses and misses were considered as no button responses when participants should have pressed a button. We performed One-Way analysis of variances (ANOVAs) on accuracy (hits, false alarms, and misses) and reaction times among stimulus types (letter, pseudoletter, red, blue, target). Tukey-Kramer *post-hoc* tests were performed on significant ANOVA effects. Statistical results were considered significant at *p* < 0.05.

#### Event-related potentials (ERPs)

ERPs were time locked to the each stimulus onset and epoched to yield trials of −500 to 1500 ms. Trials with ERPs exceeding ±100 microV between −350 and 850 ms were rejected from further analyses. We subsequently performed a principle component artifact reduction procedure with a principle component threshold of ±100 microV between −500 to 1500 ms in order to reduce the rising and falling edges of artifacts that might remain within the interval of −350 to 850 ms window (Picton et al., 2000). This ensured that the artifacts did not contaminate the prestimulus interval during baseline correction between −200 to 0 ms. The mean, standard deviation, and range (in parentheses) for artefact-free trials for each Task-Stimulus type are as follow: Orthography-Letters = 125 ± 36 (42–172); Orthography-Pseudoletters = 125 ± 35 (44–159); Color-Letters = 117 ± 41 (45–158); Color-Pseudoletters = 130 ± 49 (43–182); Target-Letters = 122 ± 26 (42–153); Target-Pseudoletters = 125 ± 17 (87–145); and Target-Targets = 47 ± 13 (20–69). Artifact-free trials were averaged across trials and filtered using a 30-Hz low-pass filter to obtain evoked potentials (EPs) for each stimulus type (letters and pseudoletters) within each task condition (Orthography, Color, and Target). For the purpose of this study, we only investigated the EPs to letters and pseudoletters among tasks. Target stimuli (numbers 1 and 2) were excluded from our analyses and results. We also calculated the global field power (GFP) as the root-mean-squared values of the EPs averaged across the scalp electrodes (excluding the electrooculogram electrodes) for each sample.

We performed Two-Way ANOVAs on the EP and GFP waveforms averaged over 25 ms intervals spanning from −100 to 600 ms across Tasks (Orthography, Color, and Target) and Stimulus type (letter and pseudoletter). Main effects and interactions were considered significant at *p* < 0.05. Tukey-Kramer *post-hoc* tests were performed on significant ANOVA main effects of Task. *Post-hoc* results were considered significant at *p* < 0.05. We also evaluated ANOVA and *post-hoc* results at significance levels of *p* < 0.01 and *p* < 0.001.

In addition to statistical testing across samples, we performed statistical analyses on the P1, N1, and P2 peak amplitudes and latencies at electrodes PO9h, PO10h, P7, and P8. These electrode sites were chosen because they had significant Stimulus effects from the Two-Way ANOVAs described above. An experienced rater manually identified peak responses with a maximum between 50–100 ms as P1, a first minimum between 50–250 ms as N1, and a maximum between 150–300 ms as P2 for electrodes PO9h, PO10h, P7, and P8. In addition, P3 peaks were identified in electrode Pz as a maximum between 200 and 600 ms. Three-Way ANOVAs were performed for peak amplitudes and latencies for the P1, N1, P2, and P3 peaks across stimulus type (letter and pseudoletter), tasks (Orthography, Color, and Target) and hemisphere (left hemisphere = averaged PO9h and P7; right hemisphere = averaged PO10h and P8).

#### Dipole modeling

Dipole modeling using BESA software (BESA GmbH; www.besa.de) was performed *post-hoc* on EP difference waveforms for significant main effects of Task (Orthography, Color, Target) and Stimulus (letter vs. pseudoletter). This was done to determine the possible source locations of processing differences between Tasks and Stimulus types. For the Task-effects model, a pair of symmetrically-constrained dipoles was fitted to significant differences that occurred between 175 and 200 ms for the Orthography vs. Target and Color vs. Target contrasts (i.e., a selection negativity component). A third dipole was fitted to the significant differences between 225 and 250 ms for the Color vs. Target contrast (i.e., an N2 component). A fourth dipole was fitted to the significant differences between 300 and 500 ms for the Orthography vs. Target and Color vs. Target contrasts (i.e., a P3 component). Residual variances for the source modeling of the difference waves were less than 10% for all intervals. Talairach locations for these dipoles were *x* = ± 45.5, *y* = −56.0, *z* = −17.2 mm (left/right fusiform gyri); *x* = 4.1, *y* = 2.9, *z* = 49.9 mm (medial frontal gyrus); and *x* = −3.6, *y* = −61.0, *z* = 5.3 mm (lyngual gyrus). For the Stimulus-effects model, two pairs of symmetrically constrained dipoles were used to model the significant differences occurring between 150–200 ms (around the N1 peak) and between 225–300 ms (around the P2 peak). Residual variances for the source modeling of the difference waves (letter minus pseudoletter) were less than 10% for both intervals. Talairach locations for these dipoles were *x* = ± 42.6, *y* = −72.4, and *z* = −14.4 mm (left/right fusiform gyri); and *x* = ± 41.4, *y* = −62.1, and *z* = −0.6 mm (left/right inferior temporal gyri).

Similar to the statistical analyses used for the EP waveforms, we performed Two-Way ANOVAs on the dipole waveforms averaged over 25 ms intervals spanning from −100 to 600 ms across Tasks (Orthography, Color, and target) and Stimulus type (letter and pseudoletter). This was done for both the dipole models of EP difference waveforms for the Task and Stimulus effects. Tukey-Kramer *post-hoc* tests were performed on the significant ANOVA main effects of Task. ANOVA and *post-hoc t*-test results were considered significant at *p* < 0.05. We also evaluated ANOVA and *post-hoc* results at significance levels of *p* < 0.01 and *p* < 0.001.

## Results

### Behavioral responses

Behavioral responses showed participants were highly accurate at discriminating among stimuli and detecting targets (see Table 1). However, ANOVA and Tukey-Kramer *post-hoc* testing revealed that participants were less accurate at pressing the correct button to red stimuli in the Color task than to any other stimuli across tasks (see Table 1 for means; *F* = 7.2; *df* = 4, 50; *p* = 0.0001). This was a result of making more false alarms to red stimuli as compared to other stimuli (see Table 1 for means; *F* = 14.1; *df* = 4, 50; *p* < 0.0001) and not misses (*F* = 0.56; *df* = 4, 50; *p* = 0.6897). ANOVA results for RTs did not support significant differences in RTs among stimulus type (letter, pseudoletter, red, blue, target) (see Table 1 for means; *F* = 2.52; *df* = 4, 50; *p* = 0.0526). Although the ANOVA results for RTs were close to significance, this was driven by reaction times to targets being most delayed as compared to the other stimulus types (see Table 1).

**Table 1:** **Behavioral results**.

	**Orthography**		**Color**		**Target**
	**Letter**	**Pseudoletter**	**Red**	**Blue**	**Numbers**
Hits (%)	94.9 ± 3.5	94.7 ± 3.1	90.3 ± 2.7	97.1 ± 3.4	97.4 ± 4.7
False alarms (%)	3.2 ± 1.5	3.6 ± 2.5	8.3 ± 3.0	2.2 ± 2.2	0.4 ± 0.5
Misses (%)	1.9 ± 3.1	1.6 ± 3.4	1.3 ± 2.0	0.8 ± 1.5	2.6 ± 4.7
Reaction Times (ms)	474 ± 69	482 ± 71	452 ± 106	446 ± 101	552 ± 98

### GFP and EP waveforms

**Figure 2:**
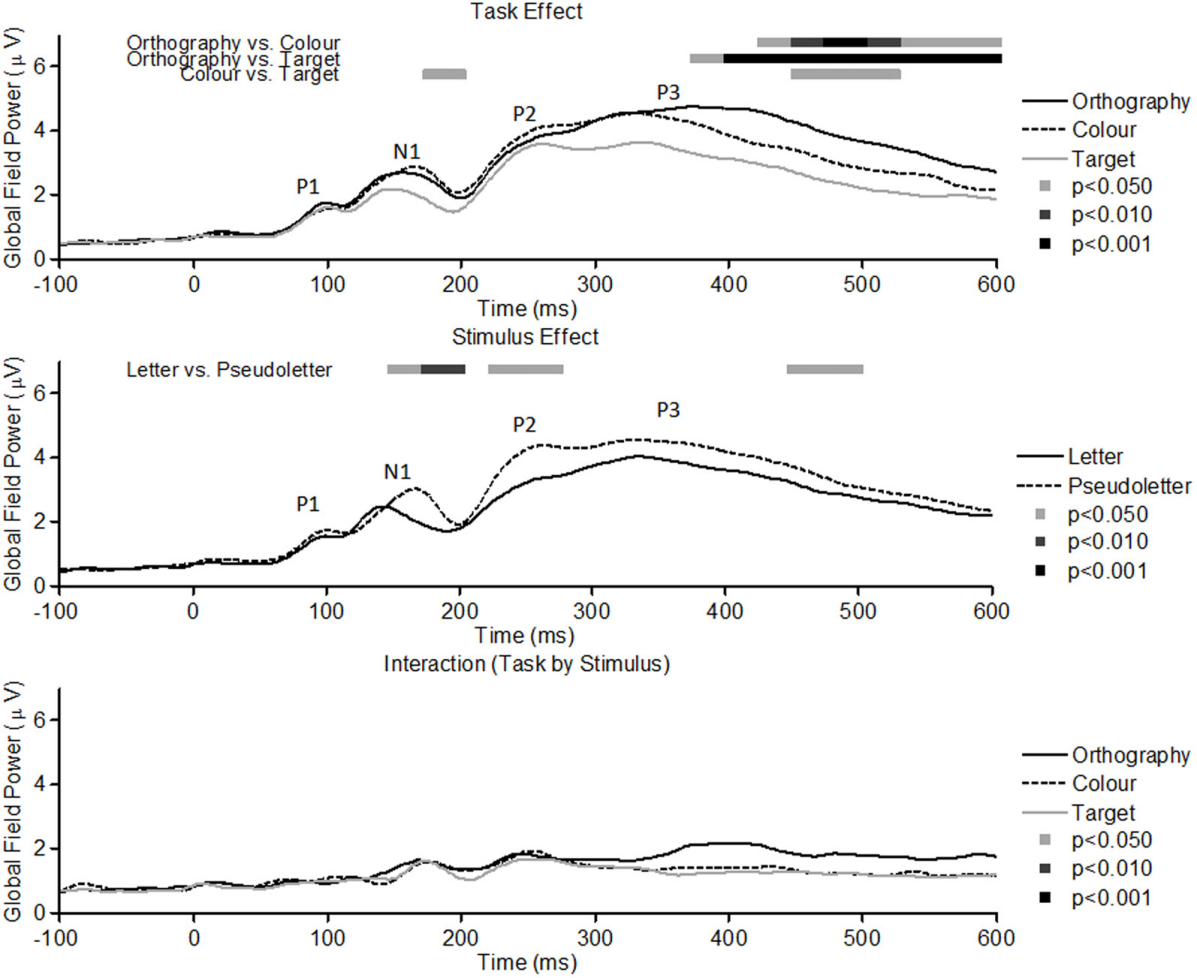
**Global field power of evoked potentials for Task effects (top plots), Stimulus effects (middle plots), and Interaction of Task by Stimulus (bottom plots).** Waveforms for the Task effect are averaged across stimulus type (letters and pseudoletters) and waveforms for the Stimulus effect are averaged across tasks (Orthography, Color, and Target). Waveforms for the Interaction are plotted as the differences between letter and pseudoletters for each task (Orthography, Color, and Target). Peaks in the waveforms reflect P1, N1, P2, and P3 responses of the evoked potentials. Bars above the waveforms designate intervals of significant main effects at *p* < 0.05, *p* < 0.01, and *p* < 0.001.

GFP waveforms showed typical responses patterns of P1, N1, P2, and P3 peaks to visual stimuli (Figure 2, top graph). Comparisons across Task (Orthography, Color, and Target) revealed that GFPs between 175 and 200 ms were significantly (*p* < 0.05) greater for Color vs. Target task and close to being significantly greater (*p* = 0.089) for the Orthography vs. Target task. GFPs between 375 and 600 ms were significantly greater for the Orthography task as compared to the Color and Target tasks. GFPs between 450 and 525 ms were significantly greater for the Color task as compared to the Target task. For the Stimulus effects, GFPs between 150–200 ms, 225–275 ms, and 450–500 ms were significantly greater for pseudoletter than letter stimuli (Figure 2, middle graph). There were no significant interactions of Task by Stimulus on GFP (Figure 2, bottom graph).

**Figure 3:**
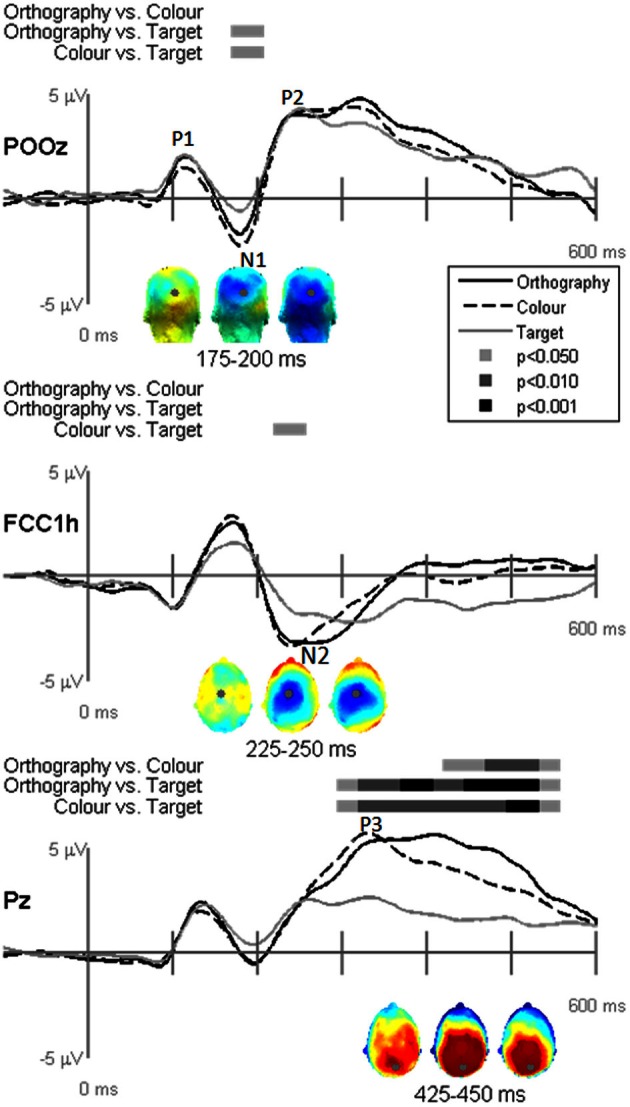
**Grand-mean evoked potentials for Task effects averaged across stimulus type (letters and pseudoletters) at electrodes POz, FCC1h, and Pz.** Bars above the waveforms designate intervals of significant differences between task comparisons at *p* < 0.05, *p* < 0.01, and *p* < 0.001. Scalp topographies plotted under the waveforms reflect the task contrasts of Orthography vs. Color, Orthography vs. Target, and Color vs. Target going from left to right. The topographies for the Task effects between 175–200 ms are shown for a posterior view and the topographies for the Task effects between 225–250 ms and 425–450 ms are shown for a top view (nose pointing to top of page). The gray dots in the topographies reflect the electrode location for the waveforms plotted above.

**Figure 4:**
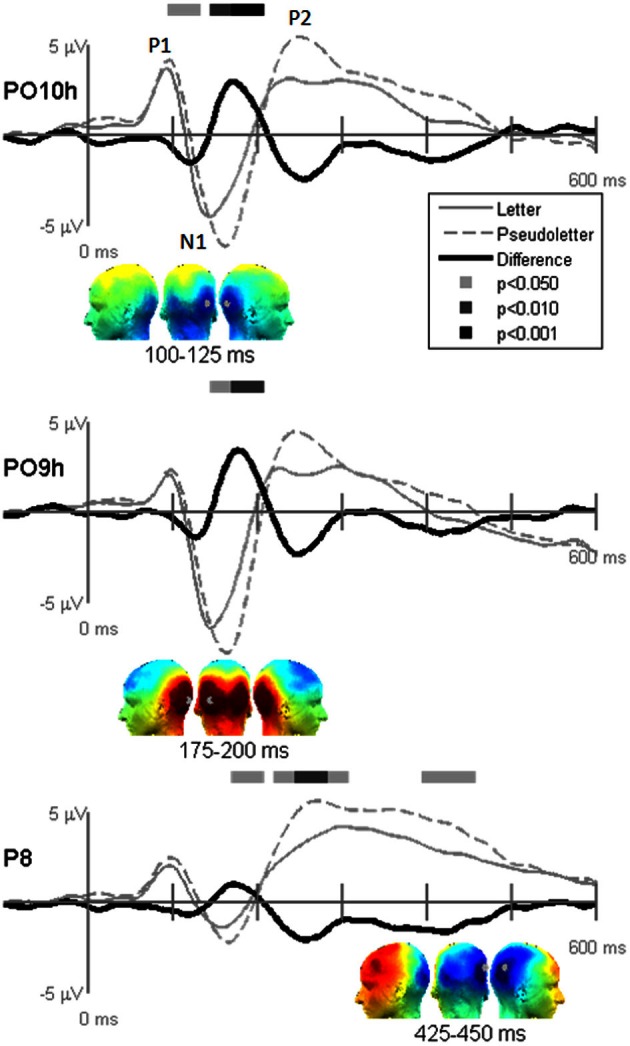
**Grand-mean evoked potentials for Stimulus effects averaged across tasks (Orthography, Color, and Target) at electrodes PO10h, PO9h, and P6.** Bars above the waveforms designate intervals of significant differences between letters and pseudoletters at *p* < 0.05, *p* < 0.01, and *p* < 0.001. Scalp topographies plotted under the waveforms reflect difference waveforms averaged across the designated intervals shown for left, posterior, and right views. The gray dots in the topographies reflect the electrode location for the waveforms plotted above.

EP waveforms showed typical P1-N1-P2 responses to the letter and pseudoletter stimuli (Figures 3 and 4). Because participants were asked to attend to and press buttons to letter and pseudoletter stimuli in Orthography and Color tasks, additional attention-related EP responses (N2 and P3) occurred as compared to the Target task in which participants disregarded the letter and pseudoletter stimuli. In addition, Orthography and Color tasks evoked a significantly greater negative response between 175 and 200 ms (around the N1) as compared to the Target task at POz (Figure 3, top graph). Topographies of the differences among Tasks revealed that the greater negativity has a posterior scalp distribution for the Orthography vs. Target and Color vs. Target contrasts. This has a similar posterior scalp distribution and timing as an SN response that has been previously reported (Hillyard and Anllo-Vento, 1998). At central electrode sites (e.g., FCCh1), EPs were significantly greater between 225 and 250 ms for Color vs. Target task (Figure 3, middle graph). Scalp topography for this contrast revealed a central distribution of this negativity, stereotypical of an N2b component. Although the Orthography vs. Target contrast did not reach statistical significance at *p* < 0.05, *p*-value for this contrast between 225 and 250 ms was 0.09 and its topography was strikingly similar to the Color vs. Target topography. Significant EP differences among Tasks were evident at Pz spanning 300 and 550 ms (Figure 3, bottom graph). Similar to the GFP results, EPs at Pz in this interval were greatest for the Orthography task, next for the Color task, and then for the Target task. The topographies between 425 and 450 ms among the Task contrasts showed typical P3 scalp distributions with peak responses occurring over parietal regions (Figure 3, bottom topographies).

Stimulus comparison results showed that pseudoletters evoked greater and later peaking N1 waves between 100 and 200 ms than did letters (Figure 4). The significant difference in the 100–125 ms interval appeared to result from a delayed N1 onset to pseudoletters than to letters. In addition to these differences in the N1 interval, P2 responses peaking around 250 ms were greater to pseudoletters than to letters over parietal sites (e.g., P6), with a right hemispheric dominance. Topographies revealed that the significant N1 and P2 differences were mainly recorded over the parieto-occipital scalp.

**Figure 5:**
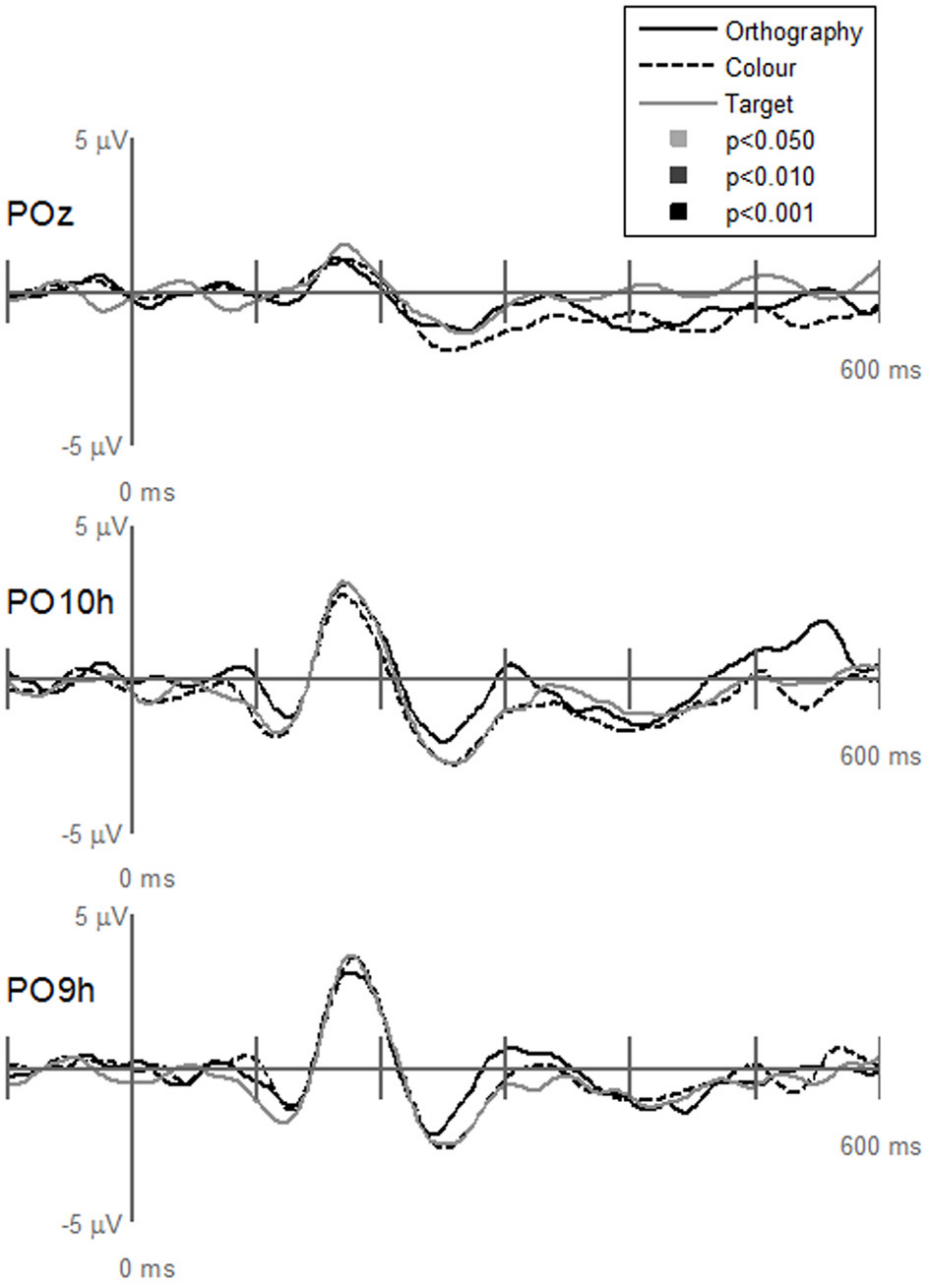
**Grand-mean evoked potentials for the Interaction of Task by Stimulus at electrodes POz, PO10h, and PO9h that showed significant Task or Stimulus effects (see Figures 3 and 4).** Waveforms are plotted as the differences between letter and pseudoletters for each task (Orthography, Color, and Target). No statistical evidence of significant interactions were found at these electrodes or at any other scalp electrodes (data not shown) at *p* < 0.05, *p* < 0.01, and *p* < 0.001.

Contrary to our hypothesis that the N1and P2 responses differences between letters and pseudoletters would be reduced when attention was drawn away from categorizing stimuli, we found no statistical support for interactions of Task by Stimulus at electrode sites (PO10h, PO9h, and POz), which clearly showed significant main effects of Task or Stimulus (Figure 5). All tasks showed the same difference waves between letters and pseudoletters. Additionally, none of the other scalp recordings revealed significant interactions (data not shown). To further support these findings we calculated peak amplitudes and latencies for the P1, N1, P2, and P3 responses. These are shown in Tables 2 and 3 and presented below with ANOVA results.

**Table 2:** **Peak EP amplitudes**.

	**Orthography task**		**Color task**		**Target task**	
**ERP peak**	**Letter**	**Pseudoletter**	**Letter**	**Pseudoletter**	**Letter**	**Pseudoletter**
P1 LH	2.18 ± 1.91	2.51 ± 2.18	1.97 ± 1.46	1.56 ± 2.51	1.84 ± 1.19	2.55 ± 1.60
P1 RH	4.17 ± 2.3	4.03 ± 2.58	2.66 ± 1.52	3.56 ± 1.59	3.20 ± 2.35	3.91 ± 2.19
N1 LH	−6.87 ± 2.97	−7.93 ± 2.98	−7.06 ± 2.73	−8.12 ± 2.90	−5.61 ± 2.88	−6.69 ± 2.38
N1 RH	−4.90 ± 3.13	−6.14 ± 3.11	−4.48 ± 2.48	−5.85 ± 2.63	−3.84 ± 3.41	−5.18 ± 2.90
P2 LH	4.04 ± 4.22	4.92 ± 3.45	3.51 ± 2.92	5.14 ± 3.95	4.25 ± 2.85	5.54 ± 3.03
P2 RH	5.11 ± 4.03	6.18 ± 3.37	4.17 ± 3.85	6.76 ± 5.13	4.08 ± 2.54	6.02 ± 3.20
P3 @ Pz	6.28 ± 3.36	6.83 ± 2.99	5.78 ± 3.7	6.39 ± 3.51	3.12 ± 2.73	3.28 ± 2.67

**Table 3:** **Peak EP latencies**.

	**Orthography task**		**Color task**		**Target task**	
**ERP peak**	**Letter**	**Pseudoletter**	**Letter**	**Pseudoletter**	**Letter**	**Pseudoletter**
P1 LH	96 ± 8	99 ± 8	99 ± 11	101 ± 12	102 ± 14	102 ± 8
P1 RH	94 ± 6	96 ± 8	93 ± 6	97 ± 7	96 ± 9	101 ± 14
N1 LH	153 ± 17	163 ± 12	152 ± 15	165 ± 15	152 ± 19	164 ± 14
N1RH	146 ± 15	163 ± 11	148 ± 18	166 ± 12	150 ± 19	169 ± 14
P2 LH	239 ± 34	252 ± 26	239 ± 29	250 ± 26	250 ± 39	254 ± 33
P2 RH	249 ± 44	263 ± 37	239 ± 27	254 ± 24	260 ± 40	272 ± 36
P3 @ Pz	385 ± 55	409 ± 55	336 ± 37	331 ± 23	352 ± 69	331 ± 64

#### P1 peak responses

Peak P1 amplitudes averaged across tasks and stimulus types were significantly larger in the right hemisphere (averaged across P8 and PO10h electrodes; 3.61 ± 2.11 μV) than the left hemisphere (averaged across P7 and PO9h electrodes; 2.11 ± 1.83 μV) (*F* = 16.96; *df* = 1, 112; *p* < 0.0001). No other ANOVA effects or interactions for P1 amplitudes were found to be significant (*p* > 0.20). A significant ANOVA hemispheric effect for P1 latencies revealed P1 peaked earlier in the right (96 ± 10 ms) than left hemisphere (100 ± 9 ms) (*F* = 4.59; *df* = 1, 112; *p* = 0.0343). No other ANOVA effects or interactions for P1 latencies were found to be significant (*p* > 0.17).

#### N1 peak responses

Peak N1 responses were significantly larger to pseudoletters (−6.66 ± 2.92 μV) than to letters (−5.47 ± 3.07 μV) (*F* = 5.213; *df* = 1, 112; *p* = 0.0243). ANOVA results also revealed a significant hemispheric effect whereby N1 amplitudes were larger in the left (−7.06 ± 2.83 μV) than right hemisphere (−5.08 ± 2.95 μV) (*F* = 14.475; *df* = 1, 112; *p* = 0.00023). No other ANOVA effects or interactions for N1 amplitudes were found to be significant (*p* > 0.15). N1 responses peaked significantly earlier to letters (150 ± 17 ms) than pseudoletters (165 ± 13 ms) (*F* = 29.419; *df* = 1, 112; *p* < 0.00001).

#### P2 peak responses

Peak P2 responses were significantly larger to pseudoletters (5.75 ± 3.64 μV) than to letters (4.21 ± 3.38 μV) (*F* = 5.801; *df* = 1, 112; *p* = 0.0177). No other ANOVA effects or interactions for P2 amplitudes were found to be significant (*p* > 0.2). Peak P2 latencies were not found to show any significant effects or interactions among task, stimulus type, and hemisphere (*p* > 0.06).

#### P3 peak responses

Peak P3 responses were significantly larger for the Orthography (6.41 ± 3.19 μV) and Color (6.06 ± 3.54 μV) tasks as separately compared to Target task (3.1 ± 2.66 μV) (*F* = 5.801; *df* = 1, 112; *p* = 0.0177). No other ANOVA effects or interactions for P3 amplitudes were found to be significant (*p* > 0.60). ANOVA and *post-hoc* testing revealed that P3 responses peaked significantly later for the Orthography task (394 ± 50 ms) as separately compared to the Color (333 ± 38 ms) and Target (344 ± 34 ms) tasks (*F* = 12.447; *df* = 2, 56; *p* < 0.0001). No other ANOVA effects or interactions for P3 latencies were found to be significant (*p* > 0.87).

### Dipole waveforms

**Figure 6:**
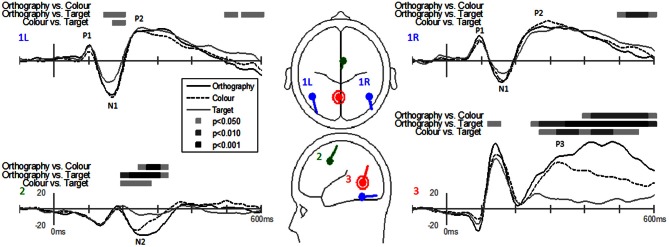
**Grand-mean source waveforms for Task effects averaged across stimulus type (letters and pseudoletters) for the Task-effects model (inset) with bilateral dipoles in the fusiform gyri (dipoles 1L and 1R), medial frontal gyrus (dipole 2), and medial lingual gyrus (dipole 3).** Bars above the waveforms designate intervals of significant differences between task comparisons at *p* < 0.05, *p* < 0.01, and *p* < 0.001. Vertical axis scale for waveform plots is in nAmp.

**Figure 7:**
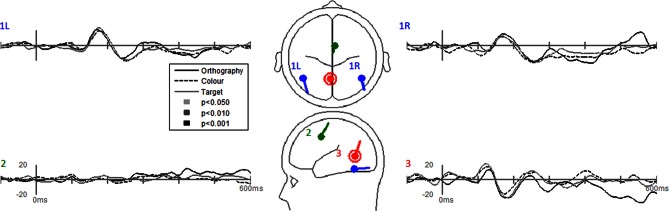
**Grand-mean source waveforms for the Interaction of Task by Stimulus for the Task-effects model (inset) with bilateral dipoles in the fusiform gyri (dipoles 1L and 1R), medial frontal gyrus (dipole 2), and medial lingual gyrus (dipole 3).** Waveforms are plotted as the differences between letter and pseudoletters for each task (Orthography, Color, and Target). No statistical evidence of significant interactions were found in these source waveforms at *p* < 0.05, *p* < 0.01, and *p* < 0.001. Vertical axis scale for waveform plots is in nAmp.

**Figure 8:**
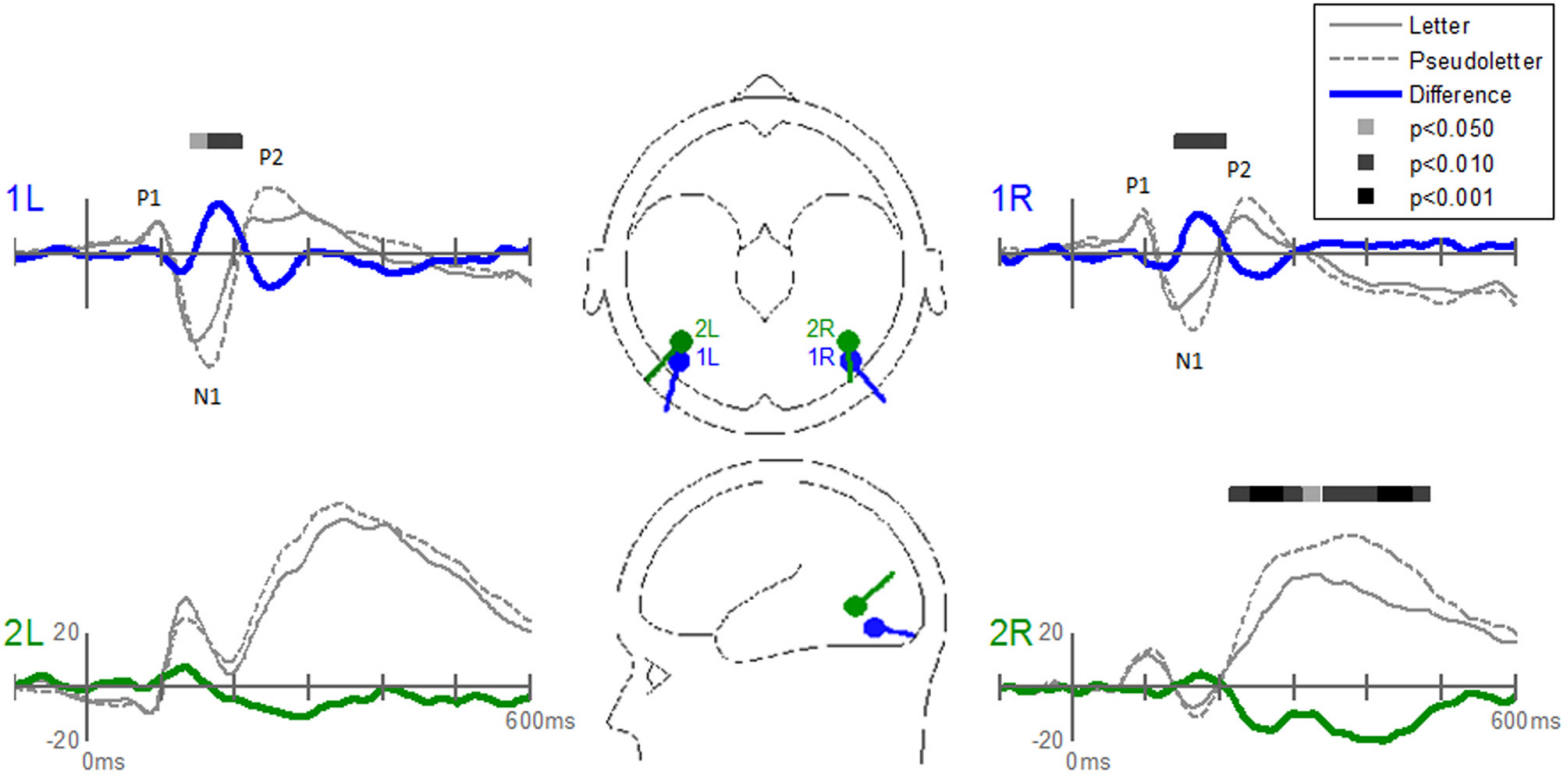
**Grand-mean source waveforms for Stimulus effects averaged across tasks (Orthography, Color, and Target) for the Stimulus-effects model (inset) with bilateral dipoles in the fusiform gyri (dipoles 1L and 1R) and bilateral inferior temporal gyri (dipoles 2L and 2R).** Bars above the waveforms designate intervals of significant differences between letters and pseudoletters at *p* < 0.05, *p* < 0.01, and *p* < 0.001. Vertical axis scale for waveform plots is in nAmp.

**Figure 9:**
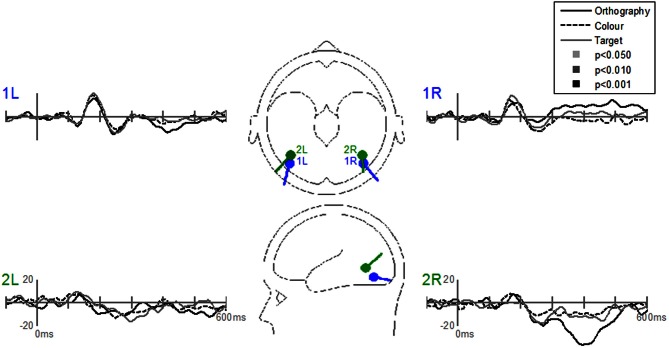
**Grand-mean source waveforms for the Interaction of Task by Stimulus for the Stimulus-effects model (inset) with bilateral dipoles in the fusiform gyri (dipoles 1L and 1R) and bilateral inferior temporal gyri (dipoles 2L and 2R).** Waveforms are plotted as the differences between letter and pseudoletters for each task (Orthography, Color, and Target). No statistical evidence of significant interactions were found in these source waveforms at *p* < 0.05, *p* < 0.01, and *p* < 0.001. Vertical axis scale for waveform plots is in nAmp.

Dipole-source waveforms showed significant effects in the Task-effects and Stimulus-effects models (Figures 6–9) similar to those seen in the EP waveforms (Figures 3–5). The Task-effects source model (Figure 6) had significantly larger N1 responses in the right fusiform gyrus (dipole 1L) for the Orthography and Color tasks as compared to the Target task. Although this effect was not significant (*p* > 0.15) in the right fusiform gyrus (dipole 1R) the waveforms showed the same larger N1 responses, as seen in the left fusiform gyrus, for the Orthography and Color tasks as compared to the Target task. The N2 effect was localized to the medial frontal gyrus (dipole 2), which showed significant N2 differences among all task contrasts. This source had a large and prolonged N2 response for the Orthography task, a smaller and narrower N2 for the Color task, and a minimally evident N2 for the Target task. Because of the prolonged nature of the N2 for the Orthography task, it was significantly larger than the N2 for the Color task. The P3 effect was localized to the midline of the lingual gyrus (dipole 4). This dipole had large responses for the Orthography and Color tasks and minimal responses for the Target task. Task contrasts revealed that the P3 response was significantly prolonged, extending out to about 500 ms, for the Orthography task as compared to the P3 response for the Color Task that peaked around 330 ms. Source waveforms for the differences between Letters and Pseudoletters for the Task-effects dipole model showed little, if any, disparity among tasks (Figure 7). Moreover, the statistical interaction of Task by Stimulus revealed no evidence that tasks modulated the responses differences between letters and pseudoletters (Figure 7).

The Stimulus-effects dipole model localized the EP differences between letters and pseudoletter to bilateral fusiform gyri (Figure 8). Source waveforms showed that bilateral fusiform gyri generated significantly larger N1 responses (between 150 and 200 ms) to pseudoletters than to letters. This is consistent with the EP results shown in Figure 4. This model further revealed that the right inferior temporal region (dipole 2R) had significantly larger P2 responses (225–325 ms) to pseudoletters than to letters. This result is consistent with the Stimulus effect shown at the P6 electrode (see Figure 4). We found no statistical evidence to support significant stimulus type differences in P2 responses in the left hemispheric source (dipole 2L). In addition, dipole 2R had significantly larger responses to pseudoletters than to letters between 350 and 475 ms. Interactions of Task by Stimulus, yet again, showed that difference waveforms (letters minus pseudoletters) showed little, if any, differences among tasks. We found no statistical evidence (i.e., no interaction of Task by Stimulus) to support the hypothesis that task modulated the differences between letters and pseudoletters (Figure 9).

## Discussion

A main finding from this study was that the early response differences between letters and pseudoletters occurring around 170 ms were not affected by task demands that encouraged attention to be directed toward (Orthography task) or away from (Color and Target tasks) orthographic stimulus features. This provides evidence that early orthographic processing of single letters is not largely influenced by selective attention to stimulus features, at least with respect to the task demands used within this study. In addition, attention did not affect the P2 differences seen in the right hemisphere. Thus, our results are in opposition to previous findings that showed attention to orthography of word stimuli enhanced early (N200) responses as compared to attention to phonology and semantics of words, which modulated later EP components (Ruz and Nobre, 2008). One explanation for our discrepant findings is that we used single character stimuli; whereas Ruz and Nobre (2008) used words and character strings. Thus, stimulus complexity and lexical retrieval might recruit higher levels of visual processes that might be influenced by top-down attention. Another difference between studies is that we used a block design for task manipulation that could have resulted in participants paying attention to letters and pseudoletters to the same degree for all tasks. However, we attempted to control for such order effects by randomly assigning task-block order across participants. Moreover, participants' attention appeared to be successfully manipulated across tasks as expected because selection negativities (SN) and N2 responses were apparent for the Orthography and Color tasks but not for the Target task (see Figures 3 and 6). The selection negativities associated with paying attention to a stimulus feature (Orthography or Color) that occurred between 175 and 200 ms had a similar scalp topography and source locations as to those shown previously (Hillyard and Anllo-Vento, 1998). In addition, the N2 following the SN had a typical topography of an attention-related N2b response, also referred to as the anterior N2 (Folstein and Van Petten, 2008). Further indication that this study's tasks modulated participants' attention was that P3 responses increased in amplitude with increasing task demands on directing attention to orthography and color (Orthography-task P3 > Color-task P3 > Target-task P3). In contrast to our study, Ruz and Nobre (2008) used a trial-to-trial cueing paradigm for drawing participants' attention to orthographic, phonologic, or semantic stimulus features. Thus, task procedures and sensory-to-motor mapping were required to be maintained throughout the block and could have recruited networks associated with perceptual and motor processes in which attention could modulate activity. Furthermore, attention effects in their study were only provided for the word stimuli and thus differences in orthographic processing between words and false-font strings are not available for comparison to the present study's results.

Another main result from this study is that we further replicated the findings that the N1 peaked earlier to letters than pseudoletters and that P2 responses are greater to pseudoletters than letters (Appelbaum et al., 2009; Herdman, 2011). These findings add support to the notion that letters are processed faster and to a lesser degree than pseudoletters. This makes sense because adult participants had many years of consolidating visual templates for familiar letters as compared to unfamiliar pseudoletters; thus template matching for letter recognition should be fairly automatic and require minimal processing. This is in line with many models of reading (e.g., McClelland and Rabinovitch, 1981; Price, 2000; Grainger et al., 2008). Contrary to our original hypothesis, task demands appeared not to affect either the early or later stages of letter and pseudoletter processing. Thus, these processes appear to be resistant to the attention demands we placed on the participants in this study and signify that letter-pseudoletter effects are most likely sensory-contingent processes, at least in adults.

Interestingly, the N1 responses and difference waveforms between letters and pseudoletters were largest in the left as compared to the right visual cortices. This is consistent with a left-lateralized language model for reading (Price et al., 2003; Cohen and Dehaene, 2004; Dehaene et al., 2005) and could be akin to the N200 effects (Nobre et al., 1994; Ruz and Nobre, 2008). However, this laterality is in opposition to a right-dominant effect showing greater processing for pseudoletters that we and others previously reported (Appelbaum et al., 2009; Herdman, 2011). Given similarities in timing, topography, and source locations across studies for the N1 letter-pseudoletter effect indicates that these are likely analogous processing effects. However, at this point we cannot explain the discrepant findings among these studies. Task differences among studies are unlikely because the current experiment found no evidence for task effects for similar tasks and stimuli to those previously used in the literature. More research is thus warranted to determine laterality of these early visual processing differences between letters and pseudoletters.

Possible explanations for the larger and later peaking N1 and the larger P2 to pseudoletters than letters is that extra processing of unfamiliar objects occurs in order to identify and categorize the unfamiliar pseudoletters (Appelbaum et al., 2009; Herdman, 2011) or that pseudoletters capture attention to a greater extent and thus modulate early visual processing (Vinckier et al., 2007; Ruz and Nobre, 2008). However, this later possibility is less likely because we found no change in letter-pseudoletter processing differences among the tasks that manipulated attention to or away from orthographic stimulus features. It appears that the different levels of attention paid to stimulus features did not alter the broader N1 and larger P2 responses to pseudoletters. Thus, the results indicate that the greater responses to pseudoletters appear to be sensory-contingent and are not under the control of attentional focus. This further leads us to believe that the N1 and P2 enhancements are likely related to the initial processing stages that are molded by experience to become more rapid and efficient at identifying letters than pseudoletters. In this case, bigger or broader is not better. Bigger responses here reveal more processing of the stimulus attributes, which requires more energy and poorer efficiency. The EPs to letters peaking earlier and with reduced neural responses, points toward consolidation of letter templates within neural ensembles to allow for rapid and accurate identification of these highly familiar letters. The finding that the behavioral reaction times are faster to letters than pseudoletters (LaBerge, 1973; Herdman, 2011; also in present study but not significant) further supports a more efficient system for processing familiar letters than unfamiliar pseudoletters.

EPs can peak later because of deconstructive addition upon averaging. Two reasons for this deconstructive addition is that there is greater variability in the timing by which neural populations are synchronously evoked by stimuli (i.e., less overlapping components of the N1) or there is greater trial-to-trial latency jitter of the EP. These would also reduce the EP amplitudes. We found that the N1 was larger and peaked later to pseudoletters than letters. Thus, a more likely alternate explanation for this later and larger N1 is a greater recruitment of neural ensembles. Because pseudoletters are less familiar and had very limited time to create well-formed templates within the visual networks, the brain likely attempts to first match the pseudoletters to letter templates. This could take a few template-matching iterations within the network and thus cause greater neural discharges over time as compared to more automatic template matching that would occur for letters. Such a notion fits with many reading models describing the early stages of orthographic processing (e.g., Dehaene et al., 2005; Grainger et al., 2008).

Our behavioral results were largely unremarkable. They showed that participants were fairly engaged in performing all tasks (>90% accuracy). Interestingly though we did not find statistical evidence for faster reaction times to letters than pseudoletters as previously reported; however the difference was in the right direction, about 8 ms faster to letters than pseudoletters (LaBerge, 1973; Herdman, 2011). This might have been due to statistical power issues of having a limited number of participants. We did; however, find an unexpected result in that participants made more false alarms to red than blue stimuli. This could be a result of an ecological effect in that red stimuli are commonly associated with the concept of “stop” and possibly this association is interacting with participants ability to discriminate and press the buttons (Elliot et al., 2007). Reaction times were similar between red and blue stimuli thus motor-response inhibition is unlikely. In hindsight, we should have used color stimuli that are not commonly associated with motor commands. We did not include false-alarm trials within the EP analyses so this unexpected result likely had little or no effect on our EP differences between letters and pseudoletters.

In conclusion, the present study's results provided further evidence that single letters are processed faster and with less neural activity than pseudoletters. Tasks encouraging participants to direct attention toward and away from orthographic stimulus features did not change the early (N1 at ~170 ms) and late (P2 at ~250 ms) processing differences between letters and pseudoletters. Thus, visual processing of single orthographic or non-orthographic characters appeared to be sensory-contingent and independent of top-down control of directing attention toward or away from orthographic stimulus features.

### Conflict of interest statement

The authors declare that the research was conducted in the absence of any commercial or financial relationships that could be construed as a potential conflict of interest.
